# Subchronic Toxicity of the New Iodine Complex in Dogs and Rats

**DOI:** 10.3389/fvets.2020.00184

**Published:** 2020-04-17

**Authors:** Rinat Islamov, Tatyana Kustova, Armen Nersesyan, Alexander Ilin

**Affiliations:** ^1^Scientific Center for Anti-infectious Drugs, Almaty, Kazakhstan; ^2^Institute of Cancer Research, Medical University of Vienna, Vienna, Austria

**Keywords:** laboratory animal, iodine, dog, rat, thyroid gland, testes, ovaries, toxicity

## Abstract

**Background:** Complexes of iodine (povidone-iodine and cadexomers) are among the most important antiseptics used in clinical and veterinary medicines. However, high local irritation activity and systemic toxicity limits their oral administration. The purpose of the study was to compare the effect of a new complex of iodine (PA, potentiator of anticancer antibiotics), in which iodine is coordinated by carbohydrates and polypeptides) on the organisms of rats and dogs treated orally with the drug for 30 days.

**Methods:** Wistar rats and Beagle dogs served as experimental animal models. Effect of PA on the animal organism was examined through the measurements of hormones level changes, hematological and clinical chemistry parameters alterations, necropsy and histological examination.

**Results:** The established maximum tolerated dose (MTD) of 2,000 mg/kg PA led to a decrease in the rate of body weight gain in male and female rats. Changes in hematological and certain biochemical parameters in rats at doses of 1,000 and 2,000 mg/kg were observed. Histological study of the thyroid gland revealed changes in the shape and size of the follicles along with colloid resorption. Administration of a half of MTD (180 mg/kg) and lower doses did not result in any change in dogs (thyroid-stimulating hormone, triiodothyronine, and thyroxine).

**Conclusions:** The results of our study show that the pathogenetic action of PA takes place along the path of induction of an inflammatory response with the development of thyrotoxicosis, rather than hypothyroidism. The mechanism of induction of an inflammatory response is also confirmed by histological studies of lesions of the thyroid gland and testes in rats (Figure S1). The no-observed-adverse-effect level (NOAEL) of PA is estimated to be 180 mg/kg (or iodine 22.8 mg/kg) in dogs, which is equivalent to 100 mg/kg (or iodine 12.3 mg/kg) in humans.

## Introduction

Iodine is used in medical practice as a highly effective antiseptic and disinfectant, especially against biofilms formed by *Staphylococcus aureus* (1, 2). Complex compounds of iodine with the polymer molecules, including polyvinylpyrrolidone (PVP), dextrin, etc., possess biocidal activity. It has also been shown that I_2_, rather than I^−^, is a potential inhibitor of carcinoma (3). It has recently been shown that molecular iodine may be useful in adjuvant therapy for breast cancer (4).

The PVP complex with triiodide [in the form of povidone-iodine (PVP-I_2_)] is most widely used in medical practice (5–7). However, PVP-I_2_ has serious shortcomings due to high oxidative activity of iodine (8). In this context, the oral administration of iodine solutions is significantly hampered. Various biocompatible polymers are used to enhance the binding of molecular iodine. They form strong complexes that can be used in medicine (9, 10).

To reduce the oxidative activity and stabilize iodine, a new complex of iodine, a potentiator of anticancer antibiotics (PA) was synthesized. It contains polysaccharides and polypeptides derived from hydrolyzed albumin which form a strong complex with iodine molecules. This makes possible to minimize the interaction of iodine with proteins present in the gastrointestinal tract, without significantly reducing pharmacological activity (11–13). It was previously shown *in vitro* and during the research on in mice that complexes of iodine with polypeptides and dextrin, in combination with doxorubicin, possess a potentiation effect (14, 15).

However, prolonged exposure to or administration of high doses of iodine chemical complexes preparations is accompanied by thyrotoxic reactions in the form of iodine-induced hypothyroidism (16). Existing data on the effect of high doses of iodine on the human body are known primarily from the described clinical cases associated with a single-dose administration of molecular iodine solutions. The administration of high doses is manifested not only through changes in hormone levels. Following excess iodine intake, including its absorption through the skin, changes occur in the electrolyte balance of the blood; also metabolic acidosis, hypernatremia, hyperosmolarity, hemolysis, and leukocytosis can be also observed (17–19).

Therefore, the aim of this study was to examine the influence of a new iodine complex compound including polysaccharides and polypeptides on the organism of rats and dogs after oral administration to define a no-observed-adverse-effect level (NOAEL). The ICH Guideline M3(R2) recommend that the repeated-dose toxicity should be studied on two mammalian species (one non-rodent). This is a reason why we chose the beagle dogs.

## Materials and Methods

### Test Substance

Physicochemical and biological properties of PA are described in the patent (13). PA is a dark brown powder with a faint odor of iodine. Concentration of molecular iodine in PA is 54.1 g/kg. It also contains auxiliary components (potassium iodide−87 g/kg, dextrin−863 g/kg and hydrolyzed bovine serum albumin 3.3 g/kg) which play important roles in the formation of the complex. The solution of PA was administered by gavage to rats at a volume of 1 ml. PA was administered to dogs in capsular form. Distilled water was a solvent of PA and it also served as a negative control.

### Animals

All animal studies were conducted in accordance with the procedures and principles outlined in the Guide for the Care and Use of Laboratory Animals by the National Institutes of Health. The studies were approved by the Ethics Committee of the Scientific Center (Protocol No. 18.12/07/2012). Twelve male and twelve female Wistar rats of specific pathogen free (about 5 weeks old) were obtained from the Kazakh Scientific Center for Quarantine and Zoonotic Infections (Almaty, Kazakhstan).

The rats were housed in individually ventilated cages (IVC) (Tecniplast, Italy) under controlled conditions (with a day/night cycle of 12 h, at a temperature of 22°C and relative humidity of 55%). The animals were fed with the commercial complete feed (Assortiment Agro Ltd, Russia). After 2 weeks of acclimatization, the animals were randomized into groups. Access of animals to food and water was *ad libitum*.

The number of dogs in the experiment was 32. Sixteen male and sixteen female Beagle breed dogs aged 4 to 9 months were obtained from the kennel in Konárovice (Pod Zámkem 279, Czech Republic). The animals were housed in individual steel cages with constant environmental monitoring at 15–21°C and relative humidity of about 55%. A standard diet was used for feeding 2 times a day (Delican extra, Czech Republic). The amount of feed to be given daily was 30 g per kg of animal weight. The quality of water and feed was regularly checked. The acclimatization period lasted 10 days.

### Experimental Design

Design of the experiments is presented in Table 1. Rats and dogs were administered PA daily for 30 days. All animals were monitored for clinical signs of poisoning. Measurement of individual weight, consumption of food and water was carried out on the weekly basis. After 30 days of administration of the test substance, all animals were euthanized. Rats were sacrificed by CO_2_ gas inhalation, dogs with an overdose intravenous injection of 20% pentobarbital solution (150 mg/kg).

**Table 1:** Design of the experiment.

**Group**	**Number of animals**		**Substance**	**Dose of PA (mg/kg)**
	**Males**	**Females**		
**Rats**				
R1	5	5	Water	0
R2	5	5	PA	500
R3	5	5	PA	1,000
R4	5	5	PA	2,000
**Dogs**				
D1	4	4	–	0
D2	4	4	PA	30
D3	4	4	PA	75
D4	4	4	PA	180

*R1—control group of rats, R2—R4—experimental groups of rats, D1—control group of dogs, D2—D4—experimental groups of dogs*.

### Dose Selection

In the pilot study (data is not shown), the maximum tolerated dose (MTD) of PA was determined to be 2,000 mg/kg for rats. In addition, the MTD and dose range for PA used in studying toxicity were preliminarily evaluated within 14 days in the pilot study (data are not shown). The MTD of PA was determined to be 350 mg/kg for male and female dogs.

### Serum Hormonal Assay

Blood samples for studying the hormones (thyroxine—T4, triiodothyronine—T3, and thyroid-stimulating hormone—TSH) were selected at the end of the entire period of PA administration. Blood was collected by cardiac puncture after rats euthanasia, and from *vena cephalica antebrachii* or *vena saphena* in dogs. The Unicel DxI 800 analyzer (Beckman Coulter, USA) was used to carry out studies on hormones in dogs. Serum levels of TSH, T3, and T4 hormones were measured by IEA (Vector-Best, Russia). For this purpose, blood samples were collected into Tapval tubes containing an anticoagulant. Plasma samples were prepared by centrifugation at 6,000 rpm for 15 min.

### Hematological Assay

Blood samples were collected before and after the administration of PA using the previously described techniques into tubes (Vacuette, Greiner Bio-One) containing tri-potassium ethylenediaminetetraacetate (K3-EDTA). Rat and dog hematological parameters, including white blood cells (WBC), lymphocytes (LYM), granulocytes (GRA), monocytes (MO), platelets (PLT), red blood cells (RBC) hemoglobin (HGB), hematocrit (HCT), were examined using the analyzer (Humacount, Human GmbH, Germany). Neutrophil granulocytes (SN) and eosinophilic granulocytes (EO) were also measured in dogs.

### Clinical Chemistry

Serum was isolated from the blood of the rats by centrifugation at 6,000 rpm for 15 min for clinical chemistry testing. Serum samples were examined on the A-25 biochemical analyzer (Biosystem, Spain). The following parameters were measured: alanine aminotransferase (ALT), aspartate aminotransferase (AST), alkaline phosphatase (ALP), bilirubin (Bbn), albumin (Alb), total protein (TP), glucose (Glu), urea (Urea), creatinine (Crea), cholesterol (Chol), and triglycerides (Tgly). To perform clinical chemistry tests in dogs, blood was collected from *vena cephalica antebrachii* or *vena saphena* into a Tapval tube without anticoagulant on the first day before and on the last day of PA administration. The following parameters were measured on the Dimension Vista analyzer (Siemens, USA): alanine aminotransferase (ALT), aspartate aminotransferase (AST), alkaline phosphatase (ALP), bilirubin (Bbn), albumin (Alb), globulins (Glo), total protein (TP) Glucose (Glu), urea (Urea), creatinine (Crea), cholesterol (Chol), and electrolytes: sodium (Na), potassium (K), and chlorides (Cl).

### Necropsy and Histology Studies

All animals were weighed and examined externally. The cranial, thoracic, and abdominal cavities were opened for macroscopy. The brain, heart, liver, thymus, kidneys, adrenals, spleen, testes or ovaries of dogs and rats were weighed at the end of the experiment. In addition, the dog prostate was weighed. Samples of organs and tissues were collected from each animal into a 10% buffered formalin solution (Sigma, USA). Five micrometer thick slices were prepared from the fixed samples, stained with hematoxylin-eosin, and examined under a microscope (AxioScopeA1, Carl Zeiss, Germany).

### Statistical Analysis

Mean values and standard deviations were calculated for all quantitative indices. Rat indices were subjected to statistical analysis using the Kruskal–Wallis test with a posteriori comparison of each group to the non-parametric Dunn's criterion. The data of hematological and clinical chemistry blood tests on dogs were evaluated using the non-parametric Wilcoxon test for paired samples. All calculations are carried out with the GraphPad Prism6 software (GraphPad Software, Inc., San Diego, CA, USA). The *p* < 0.05 was considered as significant difference.

## Results

### Effect of PA on the Animal Body Weight

The Tables 2, 3 show the weights of rats from group R4, in comparison with the control group R1. The weight of the rats from groups R2 and R3 did not differed from the control D1, for males and females, respectively. Tables S1, S2 shows that the body weight in dogs were no significant changes.

**Table 2:** Body weight changes in male rats.

**Dose (mg/kg)**	**Prior to administration**	**Day 7**	**Day 14**	**Day 21**	**Day 30**
Vehicle (water)	144.0 ± 1.9	164.2 ± 3.7	180.2 ± 6.0	194.2 ± 9.1	206.6 ± 8.1
PA, 500	141.8 ± 7.7	161.0 ± 9.4	178.0 ± 11.4	193.8 ± 18.0	205.6 ± 17.1
PA, 1000	137.4 ± 9.1	163.2 ± 11.5	183.8 ± 13.4	198.8 ± 20.6	211.4 ± 18.8
PA, 2000	143.8 ± 9.5	151.4 ± 10.6	161.8 ± 16.0^*^	176.8 ± 14.1^*^	187.6 ± 13.9^*^

* *Significant difference from the control group (p <0.05)*.

**Table 3:** Body weight changes in female rats.

**Dose (mg/kg)**	**Prior to administration**	**Day 7**	**Day 14**	**Day 21**	**Day 30**
Vehicle (water)	144.0 ± 7.0	167.4 ± 11.3	180.8 ± 12.3	200.0 ± 18.1	208.8 ± 18.1
PA, 500	141.2 ± 6.8	160.6 ± 3, 8	181.4 ± 7.3	199.0 ± 11.7	212.2 ± 11.5
PA, 1000	141.0 ± 2.9	168.6 ± 3.4	191.0 ± 6.0	208.4 ± 9.6	219.6 ± 13.1
PA, 2000	144.2 ± 5.5	149.0 ± 12.7	160.4 ± 6.6^**^	177.8 ± 17.2^*^	185.2 ± 7.3^*^

*, ** *Significant difference from the control group (p < 0.05) and (p < 0.01), respectively*.

### Effect of PA on the Weight of Animal Internal Organs

Administration of PA does not affect the weight of internal organs, including the testes and ovaries of rats and dogs. However, at a high dose there was a significant increase in the liver weight in male and female rats by 26% (*p* = 0.0056) and 20% (*p* = 0.034), respectively.

### Measurement of Hormone Levels

Table S3 shows the results measuring the thyroid hormones and TSH of the four groups of rats.

The serum levels of TSH, T4 and T3 in rats exposed to different doses (500, 1,000 and 2,000 mg/kg) of PA didn't show significant alterations on the after 30 day administration compared to control.

The mean values for the thyroid hormones and TSH for the four groups of dogs are presented in Table S4.

Canine thyroid hormones also did not respond to the administration of three levels of PA doses. Measurement of the TSH level in dogs was not possible due to the limited sensitivity of the method. Only one animal in D1 group had a TSH level of ≤ 0.7 mIU/l. The reference range for TSH 0.0–0.6 mg/l or 0.0–33 mIU/l, which is consistent the results achieved (20). The reference interval for total triiodothyronine and thyroxine for dogs is 0.7–2.5 and 3.86–54.1 nmol/l, respectively (21, 22). Practically our T4 data for dogs is within this range, exception for lowest values of T3.

### Hematological Studies

The data from hematological studies in rats 30 days after exposure to the test substance are summarized and presented in Tables 4, 5.

**Table 4:** Hematological parameters in male rats.

**Parameter**	**Dose (mg/kg/day)**			
	**Vehicle (water)**	**500**	**1,000**	**2,000**
WBC (×10^3^/ul)	10.51 ± 4.27	14.24 ± 1.42	14.13 ± 2.81	7.29 ± 1.34
LYM (×10^3^/ul)	6.21 ± 3.38	8.16 ± 0.87	10.76 ± 2.86	4.38 ± 1.22
MO (×10^3^/ul)	1.11 ± 0.32	2.04 ± 0.12*	1.57 ± 0.20	1.14 ± 0.44
GRA (×10^3^/ul)	3.22 ± 0.63	4.08 ± 0.57	1.80 ± 0.23^**^	1.77 ± 0.43^**^
RBC (×10^6^/ul)	9.97 ± 0.47	11.7 ± 1.86	11.50 ± 2.76	8.70 ± 1.82*
HGB (g/l)	136.40 ± 9.10	161.00 ± 12.31	145.80 ± 15.40	132.80 ± 4.87*
HCT (%)	51.40 ± 2.86	64.08 ± 9.27	57.94 ± 12.37	49.46 ± 8.22*
PLT (×10^3^/ul)	942.60 ± 107.83	758.00 ± 167.32*	932.60 ± 269.10	954.20 ± 123.66

*** *Significant difference from the control group (p < 0.05),*.

** *Significant difference from the 500 mg/kg group (p < 0.05)*.

*WBC, white blood cells; LYM, lymphocytes; MO, monocytes; GRA, granulocytes; RBC, red blood cells; HGB, hemoglobin; HCT, hematocrit; PLT, platelets*.

**Table 5:** Hematological parameters in female rats.

**Parameter**	**Dose (mg/kg/day)**			
	**Vehicle (water)**	**500**	**1,000**	**2,000**
WBC (×10^3^/ul)	10.54 ± 3.52	13.60 ± 0.29	13.22 ± 0.87^*^	7.19 ± 1.44
LYM (×10^3^/ul)	6.01 ± 2.71	7.83 ± 0.37	9.99 ± 1.05^**^	4.78 ± 1.39
MO (×10^3^/ul)	1.45 ± 0.30	1.95 ± 0.15	1.16 ± 0.21	0.94 ± 0.08^***^
GRA (×10^3^/ul)	3.12 ± 1.59	3.86 ± 0.19	2.12 ± 0.32	1.47 ± 0.06^*^
RBC (×10^6^/ul)	10.02 ± 1.83	10.84 ± 0.55	9.42 ± 0.39	9.08 ± 0.31^*^
HGB (g/l)	132.00 ± 12.55	154.00 ± 4.85^**^	136.80 ± 1.30	127.20 ± 3.70
HCT (%)	52.68 ± 5.63	59.78 ± 1.97	53.18 ± 1.75	47.16 ± 2.05^***^
PLT (×10^3^/ul)	995.80 ± 467.77	711.00 ± 50.19	1018.80 ± 185.71	1031.40 ± 119.48

*, ** *Significant difference from the control group (p < 0.05) and (p < 0.01), respectively*.

*** *Significant difference from the 500 mg/kg group (p < 0.01)*.

*WBC, white blood cells; LYM, lymphocytes; MO, monocytes; GRA, granulocytes; RBC, red blood cells; HGB, hemoglobin; HCT, hematocrit; PLT, platelets*.

After 30 days of PA administration minor changes in hematological parameters of white blood of males were found. Monocytosis was detected at a minimal dose (*p* = 0.04), whereas at mean and maximum dose levels granulocytes were reduced (*p* = 0.002). In females, there was an increase (*p* = 0.01) in the total number of leukocytes and lymphocytes only at dose of 1,000 mg/kg of PA. On the contrary, a decrease in monocyte (*p* = 0.002) and granulocyte (*p* = 0.03) count was found at a maximum dose in females. There was a significant decrease in hemoglobin and hematocrit (*p* = 0.04) in male rats at 2,000 mg/kg of PA. In this case, the hemoglobin level increased (*p* = 0.004) at 500 mg/kg PA, and levels of erythrocytes and hematocrit decreased in female rats at the maximum dose (*p* = 0.03 and *p* = 0.002, respectively). In addition, there was a drop in platelet count at the minimal dose (*p* = 0.04) in male rats.

Tables S5, S6 shows the results of hematology parameters in dogs. No statistically significant differences were detected among any of the groups.

### Clinical Chemistry

After subchronic exposure of rats to PA, a significant elevation of ALT and AST enzymes was observed at a dose of 2,000 mg/kg PA in males, (*p* = 0.003 and *p* = 0.0129, respectively) (Table 6). In this case, in females (Table 7), ALT level was significantly elevated at 1,000 and 2,000 mg/kg PA (*p* = 0.0041). A high AST level was observed only at the highest dose of PA (*p* = 0.0018). There was a dose-dependent increase in ALP with a significance level in male (*p* = 0.01) and female rats (*p* = 0.0078). Significant (*p* = 0.028) increase in bilirubin was recorded in females at 1,000 mg/kg PA, but actually in comparison with the group after exposure to 500 mg/kg PA. Dose-dependent decrease in albumin is indicated only for males at the minimum and maximum dose of PA (*p* = 0.02 and *p* = 0.0012). The increased level of total protein level was observed in both genders (*p* = 0.03). It should be noted that an increase in the products of the metabolism of amino acids and proteins, urea and creatinine was recorded at the average and maximum dose with the significance level for males and females (*p* = 0.02 and *p* = 0.0023, respectively) against the 500 mg/kg group (*p* = 0.001). It should be noted that some biomarkers of lipid metabolism such as triglycerides and cholesterol, were not changed. No significant alterations were found in the levels of glucose and bilirubin in male rats, albumin and glucose in females.

**Table 6:** Clinical chemistry parameters in serum of male rats.

**Parameter**	**Dose, mg/kg**			
	**Vehicle (water)**	**500**	**1,000**	**2,000**
ALT, U/l	79.74 ± 25.18	55.67 ± 15.25	132.74 ± 50.67	162.14 ± 18.88^*^
AST, U/l	104.70 ± 17.17	101.08 ± 5.44	163.08 ± 84.80	282.42 ± 25.94^*^
ALP, U/l	125.84 ± 35.09	143.68 ± 52.86	283.36 ± 129.97^*^	205.70 ± 5.95^*^
Bbn, umol/l	2.30 ± 1.45	0.82 ± 0.45	3.82 ± 1.02	3.49 ± 1.76
Alb, g/l	33.18 ± 7.45	17.43 ± 2.61^*^	25.15 ± 1.05	20.88 ± 1.99^**^
TP, g/l	63.29 ± 8.01	75.15 ± 10.36	70.14 ± 2.07	87.17 ± 5.24^*^
Glu, mmol/l	8.40 ± 1.69	9.82 ± 2.69	8.36 ± 0.98	8.88 ± 2.24
Urea, mmol/l	5.11 ± 2.04	5.43 ± 0.40	7.26 ± 2.22	9.32 ± 1.19^*^
Crea., umol/l	44.23 ± 3.10	42.93 ± 11.33	147.62 ± 67.78^*^	168.70 ± 12.87^*^
Chol., mmol/l	0.88 ± 0.09	1.19 ± 0.19	1.03 ± 0.10	0.83 ± 0.09
Tgly, mmol/l	0.75 ± 0.17	0.97 ± 0.16	0.65 ± 0.32	0.92 ± 0.04

*, ** *Significant difference from the control group (p < 0.05) and (p < 0.001), respectively*.

*ALT, alanine aminotransferase; AST, aspartate aminotransferase; ALP alkaline phosphatase; Bbn, bilirubin; Alb, albumin; TP, total protein; Glu, glucose; Urea, urea; Crea, creatinine; Chol, cholesterol; Tgly, triglycerides*.

**Table 7:** Clinical chemistry parameters in serum of female rats.

**Parameter**	**Dose, mg/kg**			
	**Vehicle (water)**	**500**	**1,000**	**2,000**
ALT, U/l	80.14 ± 25.73	58.64 ± 13.49	123.31 ± 30.01^*^	141.80 ± 30.40^*^
AST, U/l	106.62 ± 18.51	99.14 ± 9.10	254.74 ± 55.46	315.30 ± 47.13^*^
ALP, U/l	119.44 ± 23.90	144.86 ± 46.64^*^	249.50 ± 71.04^*^	209.56 ± 3.98^*^
Bbn, umol/l	2.36 ± 1.35	1.56 ± 1.14	4.20 ± 0.41^*^	2.64 ± 1.52
Alb, g/l	32.88 ± 8.49	29.70 ± 2.79	25.41 ± 6.26	22.71 ± 4.77
TP, g/l	63.78 ± 9.77	74.98 ± 5.86	76.95 ± 7.96	84.71 ± 7.57^*^
Glu, mmol/l	8.92 ± 1.19	9.12 ± 3.04	7.94 ± 2.71	8.82 ± 1.68
Urea, mmol/l	5.07 ± 2.28	5.23 ± 0.29	11.41 ± 2.31^*^	9.43 ± 1.62^*^
Crea., umol/l	53.70 ± 12.34	43.18 ± 10.56	194.56 ± 33.95*^*^	177.05 ± 15.58*^*^
Chol., mmol/l	0.92 ± 0.17	1.01 ± 0.17	0.80 ± 0.11	0.89 ± 0.12
Tgly, mmol/l	0.74 ± 0.16	0.98 ± 0.19	0.82 ± 0.11	0.88 ± 0.10

*, ** *Significant difference from the control group (p < 0.05) and (p < 0.001), respectively*.

*ALT, alanine aminotransferase; AST, aspartate aminotransferase; ALP alkaline phosphatase; Bbn, bilirubin; Alb, albumin; TP, total protein; Glu, glucose; Urea, urea; Crea, creatinine; Chol, cholesterol; Tgly, triglycerides*.

Tables S7, S8 show the biochemical parameters that were measured from the serum obtained from the dogs that were subchronic exposed to the evaluated complex iodine—PA. Clinical chemistry parameters did not change in all groups of dogs. Electrolyte balance was not violated.

After 30 days exposure, the experimental groups did not show any significant differences compared to the dogs in the control group.

### Necropsy and Histology Study

Gross necropsy examination of the dogs from all the groups did not find any pathological changes.

**Figure 1:**
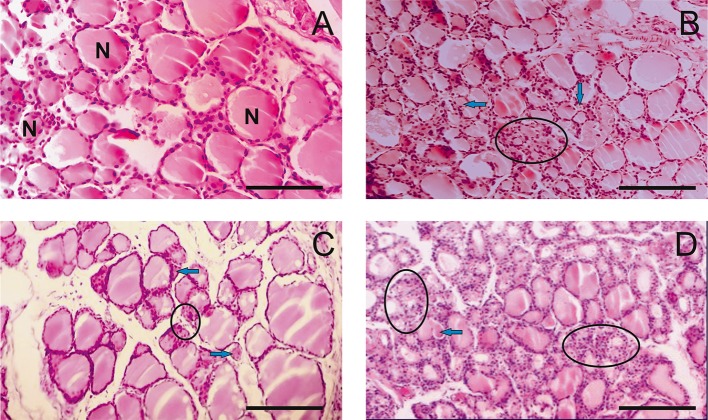
Light microscopy images of the thyroid gland in rats. **(A)** R1 group. Normal tissue. **(B–D)** R2-R4 groups. Hypothyroid tissue with variable form of thyroid follicles. The follicular thyrocytes had hypertrophy and hyperplasia. N—normal follicle with colloid, arrows—reduced form follicles, ellipse—the follicles exhausted colloid surrounded by thyrocytes with hyperplasia. (hematoxylin-eosin). Scale bar: 100 μm.

Histological examination of rat thyroid tissues revealed the pathogenetic effect of PA at doses of 500 mg/kg and higher (Figure 1).

The pathogenetic effect was characterized by the heterogeneity of the follicles with a rounded, irregular, and reduced form (Figures 1C,D). Thyrocytes were freely located in the follicular cavity, and lost contact with the basement membrane (Figure 1D). The apical parts of thyrocytes with cytoplasmic outgrowths were turned into the cavity of the follicle. Proliferative activity of thyrocytes is observed. Thyrocyte nuclei were reduced in size. Individual follicles were atrophically altered. Colloid resorption was recorded. Vessels of the capsule and partitions were enlarged.

**Figure 2:**
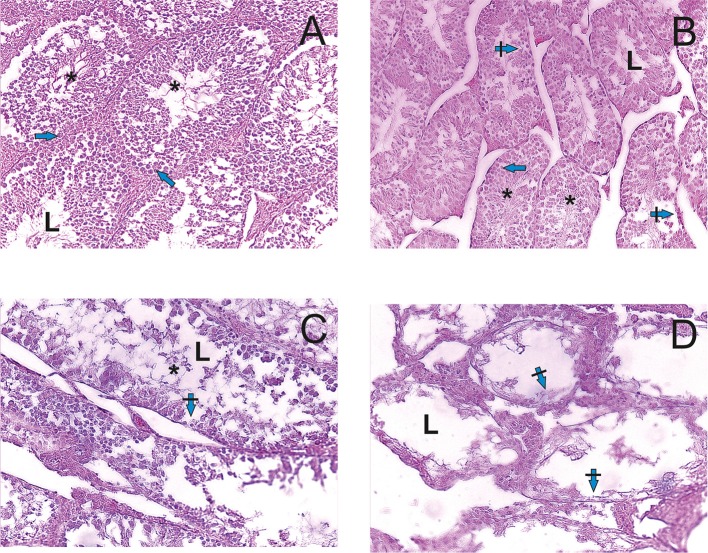
Light microscopy images of testicular tissue in rats. **(A)** R1 group. Normal testicular tissue. **(B–D)** R2-R4 groups. L—lumen, arrows—normal spermatogenic epithelium, crossed arrows—degeneration of spermatogenic epithelium, asterix—sperm cells. Edema of the interstitial tissue (hematoxylin-eosin).

Histological examination of testis (Figure 2) showed the dose dependent adverse effect of PA. The testis of R1 control animals within normal morphology showed as Figure 2A. In R2-R4 groups (Figures 2B–D) all sections of testes contained several tubules in which spermatogenesis was abnormal and more widely dilated lumen of seminiferous tubules and atrophy of the seminiferous epithelium cells are observed. There was a depletion of the cellular composition up to the basal layer. The pathologic changes of hemodynamics, phenomenona of interstitial edema and edema of the seminiferous tubules were observed. On the contrary, there were no pathologic changes in the structure of testis and prostate tissues of dogs.

**Figure 3:**
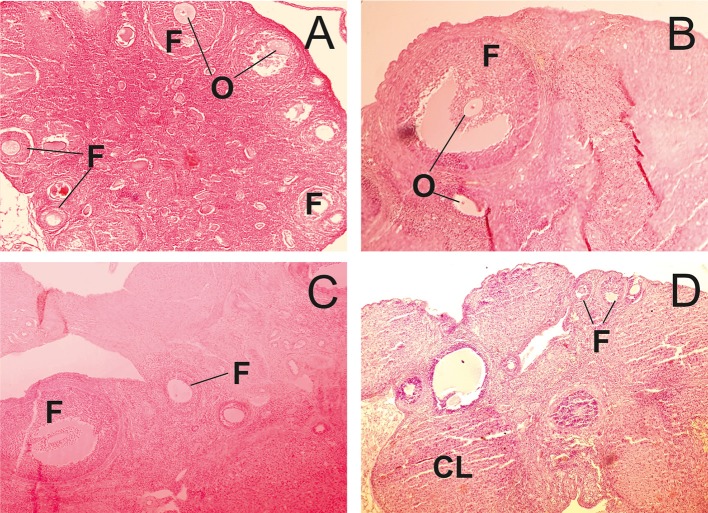
Light microscopy of ovarian tissue in rats. **(A)** R1 group. Normal ovarian tissue. **(B–D)** R2-R4 groups. F—follicles, O—oocytes, CL—corpus luteum (hematoxylin-eosin).

In the control group, the structure of ovaries in rats was within the normal range (Figure 3A). In contrast, in animals receiving of the PA, dose-dependent changes in the ovarian structure were observed (Figure 3B–D). The areas corresponding to the normal histological structure of the organ were found in the ovarian tissue at the minimum dose of PA (Figure 3B). In individual animals, single polycystic changes were identified in the yellow body. At an average dose of PA, the cortical substance predominated over the medullary substance. Cortical substance was characterized by the presence of follicles being at different stages of differentiation. However, mature follicles were not detected. A high dose of PA led to a reduction in the number of primary and secondary follicles (Figure 3D). Some animals developed ovarian endometriosis and one animal has a polycystic ovary.

**Figure 4:**
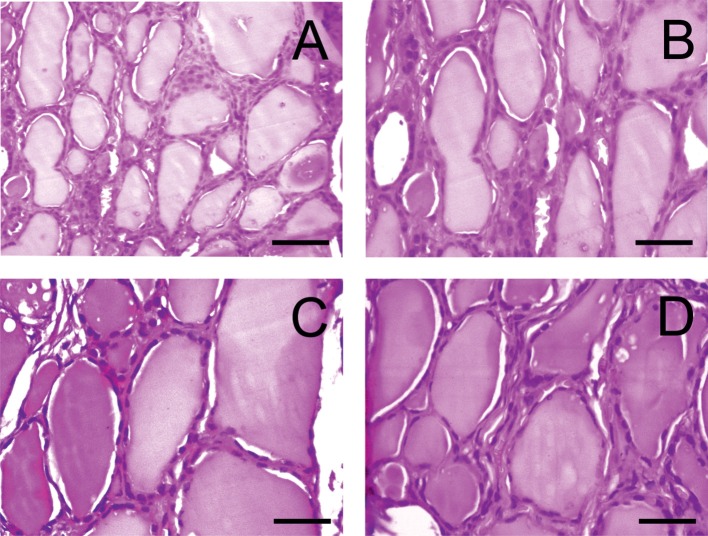
Light microscopy images of the thyroid gland in dogs. **(A)** D1 group. Normal tissue. **(B–D)** D2-D4 groups. (hematoxylin-eosin). Scale bar: 25 μm.

In opposite to rats, no pathological changes were detected in the thyroid tissues of dogs (Figure 4).

Histological analysis of control and experimental thyroids after administration of PA showed a collection of large and oval follicles composed of a monolayer of epithelial cells delineating a round lumen.

**Figure 5:**
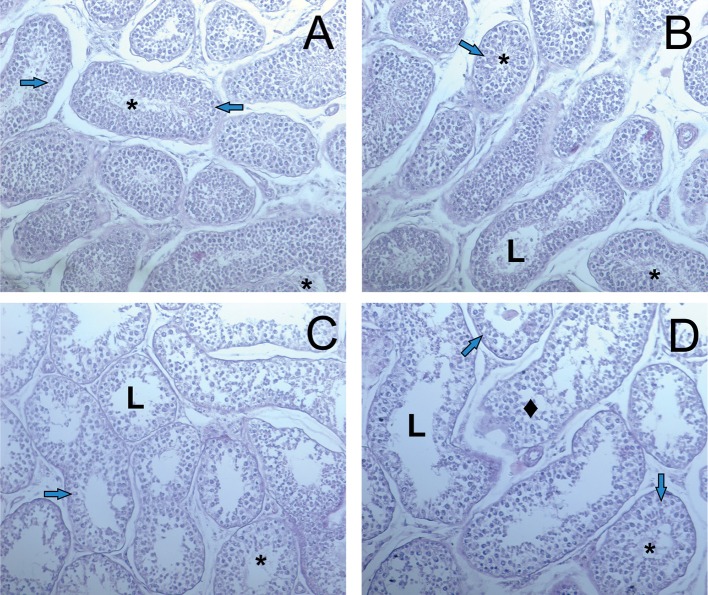
Light microscopy images of testicular tissue in dogs. **(A)** D1 group. Normal testicular tissue. **(B–D)** D2-D4 groups. L—lumen, arrows—normal spermatogenic epithelium, rhombus—degeneration of the spermatogenic cells, asterix—sperm cells. Edema of the interstitial tissue (hematoxylin-eosin).

There were minimal pathologic changes in the structure of testis of dogs (Figure 5). Only at the higher dose, sperm are rarely detected. Degenerative of the spermatogenic cells are more common.

**Figure 6:**
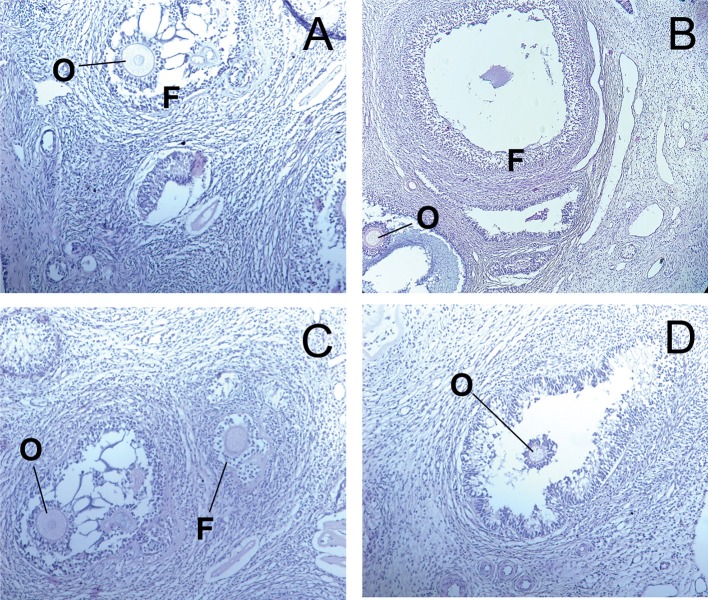
Light microscopy of ovarian tissue in dogs. **(A)** D1 group. Control normal ovarian tissue. **(B–D)** D2-D4 groups. F—follicles, O—oocytes, CL—corpus luteum (hematoxylin-eosin).

The ovaries of control and experimental dogs exhibited normal architecture, with follicles at different stages of development with oocytes exhibiting distinct nucleus (Figures 6A–D).

## Discussion

Studies on the toxicity of a PA in rats and dogs at the MTD and lower doses showed that a dose-dependent decrease in the rate of weight gain in rats at a dose of 2,000 mg/kg can be associated with decreased appetite of animals. Rats from the mean- and maximum dose groups after each administration of PA were crowded together, their motor activity decreased. After a few hours, the activity of the animals was restored. Animals lost their appetite which influenced their weight. In addition, an increase of liver mass at a dose of 2,000 mg/kg indicates a hepatotoxic effect. Iodine is characterized by such systemic toxicity (18, 23).

However, there was no reaction to the administration of iodine on the part of the TSH and thyroid hormones in rats and dogs. This can be seen in a study on the subchronic action of molecular iodine and iodides in rats at a concentration of 10 mg/l (24). This study showed that there was an increase in thyroid mass only under the action of iodides in males, and conversely, it decreased in females. On the contrary, the solution of molecular iodine did not affect the weight of the thyroid gland. At the same time, histological examination of thyroid gland also showed no pathological changes (24). Apparently, there were some differences in the bioavailability of iodine and iodides under oral administration. And most likely, this is due to the rapid iodine organification by the proteins present in the gastrointestinal tract. Adaptation of the thyroid system to high doses of iodine is also acceptable.

Weight gain dynamics in dogs of the experimental groups did not differ from the control group. Observations of dogs did not detect clinical toxicity effects.

As a rule, prolonged administration of high doses of iodine preparations may cause the appearance of an antithyroid effect in animals. However, the species sensitivity of the animals is different (23). Effect of iodine excess on the animal and human organism is related to the Wolf-Chaikoff effect which consists in a transient decrease in the level of thyroid hormones (25).

Some studies did not make it possible to determine the safe upper limit of iodine in dogs (26). Therefore, the results obtained can be of interest in terms of the effect of molecular iodine complex compounds on the dog body. A similar pattern was observed after acute administration of about 80 mg/kg of PVP-I_2_ into the pleural cavity of the adult rabbits, which also did not cause changes in T3 and T4 levels (27).

On the contrary, supplementation in the drinking water of potassium iodide at doses 3 to 24 mg/l led to a decrease in T3 and an increase in T4, as well as defects in the development of the offspring skeleton in dose-dependent manner (28). There are data confirming that the addition of 500 mg/kg iodine to a diet prior to mating did not affect the offspring survival, whereas 1,000 mg/kg caused high offspring mortality (23). All these observations confirm the importance of the normal functioning of the thyroid system in pregnant females and susceptibility of the fetuses to excess iodine.

Lack of reaction of the thyroid hormones produced by the thyroid gland to excess iodine can apparently be explained by the tolerance in an adult organism and species sensitivity to excess iodine. In addition, the different susceptibility of animals to iodine is related to the functional state of the thyroid gland and sexual specificity. When subclinical euthyroid conditions of the thyroid gland are observed, excess iodine intake can provoke thyrotoxic condition also at lower doses (29, 30). On the contrary, the normal state of the thyroid gland may provide a high tolerance to excess iodine (31).

The hematological parameters were most variable in rats. The observed increase in the levels of leukocytes and lymphocytes in the blood of females seems to indicate a non-specific inflammatory response. It was previously shown that iodine complexes stimulate the secretion of proinflammatory cytokines (6, 32). A high dose of PA, on the contrary, leads to a decrease in monocytes in females which indicates suppression of bone marrow function. Such a different effect is discussed on the example of the development of thyroid-associated lymphoid tissue ectopia in rats as a result of excess iodine intake. High doses of iodine can cause more severe autoimmune processes if the body has a tendency to dysfunction of the thyroid gland, and immune system dysregulation is observed (33). In clinical practice, agranulocytosis is observed when antithyroid drugs are administered, such as methimazole, thiourea derivatives. Moreover, drug-induced agranulocytosis can be caused by direct damaging effect on the bone marrow, immunological reactions, and free-radical processes (34).

Increase of hemoglobin in females at a dose of 500 mg/kg is possibly due to the stimulating effect of iodine, but without evident erythropoiesis. Conversely, toxic doses exclude the effect (35, 36). Reduction in platelets at a minimum dose in males is difficult to explain by the damaging effect, as for example with iodine-containing preparations such as amiodarone or iopamidol (37, 38). Moreover, this effect disappears at the maximum doses of PA. Therefore, a minor reduction in PLT count is unrelated to excess iodine intake.

At the same time, no statistically significant changes in the hematological picture of dogs were detected.

Elevated ALT and AST levels and an increase of liver mass in the group after exposure to a maximum dose of PA also indicate a hepatotoxic effect. Comparative studies undertaken by Sherer et al. (24) on the toxicity of iodine and iodides in rats at concentrations up to 100 mg/l revealed no changes in the measured parameters of clinical chemistry. Although such data are difficult to interpret due to the inability to accurately control the amount of water consumed by rats, however, the aqueous solutions of molecular iodine do not lead to changes in blood. Nevertheless, this does not exclude that higher doses may be toxic. More reliable data can be obtained in clinical cases associated with excess iodine intake. For example, postoperative treatment with large doses of povidone-iodine or its occasional ingestion leads to rapid absorption of iodides into the blood. As a result, metabolic acidosis, hypernatremia, early hyperkalemia (up to 6.6 mmol/l in the first hours) or hypokalemia (3.3 mmol/l), leukocytosis, increased creatinine, bilirubin, albumin, AST and ALT are observed (17, 19, 39, 40). The same changes were found in rats. Therefore, it could be assumed with certainty that the observed toxic effects are associated with the direct damaging action of iodine, which enters into the composition of PA. Significant changes were found in the clinical chemistry parameters of rats. The observed considerable elevation of ALP level in all experimental male and female groups indicates a direct damaging effect of PA on the tissue of gastrointestinal tract, including liver (41). At that, a statistically significant increase in bilirubin was not observed in rats, except for females which were administered 1000 mg/kg PA. On the contrary, the albumin level dropped in males, which may indicate, along with ALT, AST and ALP, the cholestatic and hepatocellular damage of the liver. The increase in TP against hypoalbuminemia at a high dose in rats is associated with inflammatory response and liver damage (42). The increased level of serum urea and creatinine is characteristic of renal damage, as was shown when iodine-containing radiopaque substances were administered (43, 44).

No significant changes of clinical chemistry parameters were revealed in dogs which may indicate the absence of damaging effects of PA in the examined doses.

Iodine-induced thyroiditis may have a localized clinical course with significant lymphocytic infiltration into the thyroid tissue and increased secretion of cytokines (45). Excess iodine causes morphological changes in the thyroid gland. There is an infiltration of macrophages into the thyroid tissue, partial disruption of the tissue architecture, reduced colloid filled with a mixture of desquamated epithelium (28, 46, 47). The observed pattern of leukocyte infiltration and partial disturbance of the colloid structure may be associated with the pathogenetic effect of PA on rat thyroid. Thereby, histological studies confirm the dose-dependent pathogenetic effect of PA on the thyroid gland.

The damaging effect of iodides after prolonged exposure to the rat testis is known and associated with the formation of reactive oxygen species (48). At the same time, the toxicity of high doses of iodides to the female mice, including pregnancy ones, is explained by thyroid dysfunction and, as a consequence, hormonal disorder (28). This can explain the dose-dependent decrease in the number of mature follicles (Figures 3B–D). However, it should also be noted that the toxicity of iodine depends on its chemical form intake. So, iodides at a dose of about 35 mg/kg for 60 days in rats cause both similar and different changes from administration of the PA iodine complex. With excessive intake of iodides in rats, body weight increases, T3 level decreases and T4 increases. Serum glucose and total cholesterol levels increase (49). In contrast, the polyiodides in PA induce a different effect on rats. But it should be mentioned that oxidative stress can be a common mechanism of cell damage (49).

Our study showed the effect of PA on the body weight of rats and dogs. At higher doses close to MTD for rats, PA caused growth impairment in animals, hematotoxic reactions, and damage to the gonads and liver in both sexes. No damaging effects were revealed in dogs.

The results obtained on the long-term systemic action of a new complex iodine compound are of considerable interest, since the toxic effect of fixed and controlled doses was first evaluated for the two species of animals. This makes extrapolation of the results to the human organism more predictable. Prior to this, the action of high iodine doses was estimated mainly based on clinical cases of a single episodic administration of high doses of iodine preparations or experiments on animals which received iodine with drinking water. It was also found that different doses of iodine relative to MTD cause more pronounced toxic reactions in rats than in dogs although it is obvious that dogs are more sensitive to an acute overdose of iodine. Whereas, dogs quickly adapt to subchronic doses of iodine. We believe that this effect is also related to effective urinary iodide excretion in dogs which significantly reduces the likelihood of damage organs (50). On the contrary, rats are not very sensitive to an acute overdose of iodine, but the long-term intake of high doses of iodine they shows the susceptibility. This is consistent with information from the review of the Committee on Minerals and Toxic Substance in Diets and Water for Animals (23) and as well as studies of Glick and colleagues with continuous the 0.5 % PVP-I irrigation of the pericardial sacs of dogs via catheters for 48 h (51).

In summary, this is the first assessment of the toxicity of molecular iodine after repeated administration on two species of animals. Our results demonstrated that MTD and its half of new complex of iodine the PA (2,000 and 1,000 mg/kg) leads to the development of thyrotoxicosis in rats, rather than hypothyroidism, as after excess intake of iodides. On the contrary, in dogs, half of the MTD is NOAEL 180 mg/kg, which corresponds for iodine is 22.8 mg/kg. Based on the formula for dose translation from animal to human (52), the human equivalent dose of iodine will be 12.3 mg/kg.

We assume that liver and gonads are the target organs due to chronic PA treatment. Probably, the damage is based on oxidative stress and acute inflammation, while changes in the ovaries of rats are associated with large fluctuations in hormone levels. It is, therefore, necessary more frequent measurement of the level of hormones which regulate ovogenesis.

## Data Availability Statement

The datasets generated for this study are available on request to the corresponding author.

## Ethics Statement

The animal study was reviewed and approved by Bioethics Commission, Scientific Center for Anti-Infectious Drugs.

## Author Contributions

RI: planning, experiment and writing of research. TK: experiment and writing of research. AN: discussion of results and review of articles. AI: discussion of results and review of articles.

## Conflict of Interest

RI, TK and AI are employed by Scientific Center for Anti-Infectious Drugs JSC. The remaining author declares that the research was conducted in the absence of any commercial or financial relationships that could be construed as a potential conflict of interest.
