# Supplementary material for: Subchronic Toxicity of the New Iodine Complex in Dogs and Rats
Source: Front Vet Sci. 2020 Apr 17;7:184. doi: 10.3389/fvets.2020.00184 (PMC7181231; doi:10.3389/fvets.2020.00184)
Supplement: Supplementary file 1 [file Table_1.DOCX]

Table S1. Body weight changes in male dogs

| **Dose (mg/kg)** | **Prior to administration** | **Day 7** | **Day 14** | **Day 21** | **Day 30** |
| --- | --- | --- | --- | --- | --- |
| Vehicle (water) | 8.26±1.50 | 8.67±1.54 | 8.92±1.54 | 8.98±1.39 | 9.09±1.37 |
| PA, 30 | 10.15±1.63 | 10.28±1.50 | 10.36±1.52 | 10.49±1.55 | 10.60±1.56 |
| PA, 75 | 8.41±0.60 | 8.51±0.66 | 8.56±0.66 | 8.63±0.69 | 8.80±0.67 |
| PA, 180 | 9.35±1.56 | 9.66±1.66 | 9.81±1.54 | 9.99±1.56 | 10.12±1.62 |
