# Supplementary material for: Subchronic Toxicity of the New Iodine Complex in Dogs and Rats
Source: Front Vet Sci. 2020 Apr 17;7:184. doi: 10.3389/fvets.2020.00184 (PMC7181231; doi:10.3389/fvets.2020.00184)
Supplement: Supplementary file 2 [file Table_2.DOCX]

Table S2. Body weight changes in female dogs

| **Dose (mg/kg)** | **Prior to administration** | **Day 7** | **Day 14** | **Day 21** | **Day 30** |
| --- | --- | --- | --- | --- | --- |
| Vehicle (water) | 8.62±1.36 | 9.17±1.39 | 9.44±1.47 | 9.65±1.53 | 9.77±1.59 |
| PA, 30 | 9.14±1.25 | 9.26±1.23 | 9.37±1.24 | 9.48±1.30 | 9.75±1.06 |
| PA, 75 | 8.64±1.50 | 8.76±1.43 | 8.83±1.43 | 8.87±1.41 | 8.96±1.42 |
| PA, 180 | 9.76±1.33 | 10.03±1.28 | 10.18±1.27 | 10.35±1.26 | 10.48±1.32 |
