# Supplementary material for: Subchronic Toxicity of the New Iodine Complex in Dogs and Rats
Source: Front Vet Sci. 2020 Apr 17;7:184. doi: 10.3389/fvets.2020.00184 (PMC7181231; doi:10.3389/fvets.2020.00184)
Supplement: Supplementary file 3 [file Table_3.DOCX]

Table S3. Hormones TSH, T3 and T4 in rats

| **Hormone** | **Sex** | **Dose (mg/kg/day)** | | | |
| --- | --- | --- | --- | --- | --- |
|  |  | **Vehicle (water)** | **500** | **1000** | **2000** |
| **TSH, (nmol/l)** | ♂ | 3.2±4.1 | 3.3±4.9 | 0.9±1.8 | 6.0±5.0 |
|  | ♀ | 0.5±0.5 | 1.1±2.3 | 1.2±1.5 | 1.9±0.8 |
| **T3, (nmol/l)** | ♂ | 9.4±1.6 | 7.5±0.7 | 7.6±0.9 | 8.5±0.9 |
|  | ♀ | 8.6±1.2 | 8.3±1.3 | 8.2±1.4 | 7.7±0.6 |
| **T4, (nmol/l)** | ♂ | 41.0±16.6 | 27.5±11.7 | 28.7±10.1 | 33.3±7.2 |
|  | ♀ | 21.5±3.3 | 25.5±8.2 | 27.2±6.1 | 27.1±3.5 |
