# Supplementary material for: Subchronic Toxicity of the New Iodine Complex in Dogs and Rats
Source: Front Vet Sci. 2020 Apr 17;7:184. doi: 10.3389/fvets.2020.00184 (PMC7181231; doi:10.3389/fvets.2020.00184)
Supplement: Supplementary file 4 [file Table_4.DOCX]

Table S4. Hormones TSH, T3 and T4 in dogs

| **Hormone** | **Sex** | **Dose (mg/kg/day)** | | | |
| --- | --- | --- | --- | --- | --- |
|  |  | **Vehicle (water)** | **30** | **75** | **180** |
| **TSH, (mlU/l)** | ♂ | <0.01 | <0.01 | <0.01 | <0.01 |
|  | ♀ | <0.01 | <0.01 | <0.01 | <0.01 |
| **T3, (nmol/l)** | ♂ | 0.76±0.06 | 0.85±0.22 | 0.52±0.12 | 0.58±0.09 |
|  | ♀ | 0.79±0.17 | 0.68±0.18 | 0.74±0.12 | 0.86±0.13 |
| **T4, (nmol/l)** | ♂ | 46.2±9.1 | 55.9±4.3 | 41.3±9.6 | 45.4±4.5 |
|  | ♀ | 43.0±8.5 | 43.1±12.1 | 55.4±10.4 | 56.8±4.8 |
