# Supplementary material for: Subchronic Toxicity of the New Iodine Complex in Dogs and Rats
Source: Front Vet Sci. 2020 Apr 17;7:184. doi: 10.3389/fvets.2020.00184 (PMC7181231; doi:10.3389/fvets.2020.00184)
Supplement: Supplementary file 5 [file Table_5.DOCX]

Table S5. Hematological parameters in male dogs

| **Parameter** | **Day** | **Dose (mg/kg/day)** | | | |
| --- | --- | --- | --- | --- | --- |
|  |  | **Vehicle (water)** | **30** | **75** | **180** |
| **WBC (x10^3^/ul)** | 0 | 10.18±1.83 | 8.75±0.79 | 8.78±0.79 | 11.08±4.77 |
|  | 30 | 9.20±1.82 | 8.28±0.49 | 8.78±0.22 | 10.13±2.03 |
| **LYM (x10^3^/ul)** | 0 | 2.15±0.73 | 2.43±0.43 | 2.28±0.45 | 2.38±1.05 |
|  | 30 | 2.23±0.63 | 1.85±0.38 | 1.85±0.38 | 2.80±0.63 |
| **MO (x10^3^/ul)** | 0 | 0.78±0.17 | 0.60±0.18 | 0.55±0.17 | 0.68±0.38 |
|  | 30 | 0.67±0.38 | 0.95±0.24 | 0.95±0.24 | 0.48±0.31 |
| **SN (x10^3^/ul)** | 0 | 5.40±1.70 | 4.90±0.70 | 4.98±0.62 | 6.62±3.80 |
|  | 30 | 4.78±1.73 | 4.80±0.84 | 4.73±0.36 | 10.32±9.43 |
| **EO (x10^3^/ul)** | 0 | 1.15±0.84 | 0.48±0.22 | 0.48±0.32 | 0.42±0.18 |
|  | 30 | 0.92±0.68 | 0.30±0.08 | 0.30±0.08 | 0.57±0.38 |
| **RBC (x10^6^/ul)** | 0 | 5.87±0.70 | 6.27±0.29 | 6.42±0.54 | 6.56±0.41 |
|  | 30 | 6.15±0.23 | 6.33±0.19 | 6.38±0.19 | 7.01±0.45 |
| **HGB (g/l)** | 0 | 136.83±15.63 | 145.75±8.81 | 148.50±6.76 | 150.95±12.33 |
|  | 30 | 141.83±4.49 | 147.50±10.08 | 144.50±8.35 | 160.52±8.98 |
| **HCT (%)** | 0 | 43.02±12.84 | 43.6±0.93 | 43.88±1.32 | 45.08±4.76 |
|  | 30 | 41.42±4.42 | 43.6±4.29 | 44.73±4.76 | 43.10±5.18 |
| **PLT (x10^3^/ul)** | 0 | 451.22±127.28 | 506±75.13 | 510.25±71.84 | 506.12±74.19 |
|  | 30 | 452.45±46.37 | 339.00±8.45 | 598.50±162.2 | 358.12±50.55 |

WBC, white blood cells; LYM, lymphocytes; MO, monocytes; SN, neutrophil granulocytes; EO, eosinophilic granulocytes RBC, red blood cells; HGB, hemoglobin; HCT, hematocrit; PLT, platelets.
