# Supplementary material for: Subchronic Toxicity of the New Iodine Complex in Dogs and Rats
Source: Front Vet Sci. 2020 Apr 17;7:184. doi: 10.3389/fvets.2020.00184 (PMC7181231; doi:10.3389/fvets.2020.00184)
Supplement: Supplementary file 6 [file Table_6.DOCX]

Table S6. Hematological parameters in female dogs

| **Parameter** | **Day** | **Dose (mg/kg/day)** | | | |
| --- | --- | --- | --- | --- | --- |
|  |  | **Vehicle (water)** | **30** | **75** | **180** |
| **WBC (x10^3^/ul)** | 0 | 10.23±1.70 | 8.73±0.98 | 8.95±1.23 | 9.63±1.10 |
|  | 30 | 9.65±1.09 | 8.55±1.52 | 9.03±1.35 | 10.2±1.48 |
| **LYM (x10^3^/ul)** | 0 | 2.63±0.82 | 2.25±0.60 | 2.3±0.48 | 2.48±0.64 |
|  | 30 | 2.65±0.55 | 1.88±0.53 | 1.78±0.46 | 2.77±0.61 |
| **MO (x10^3^/ul)** | 0 | 0.95±0.36 | 0.45±0.17 | 0.45±0.17 | 0.63±0.33 |
|  | 30 | 0.72±0.21 | 0.85±0.33 | 0.78±0.30 | 0.77±0.29 |
| **SN (x10^3^/ul)** | 0 | 5.50±1.31 | 5.23±0.92 | 4.78±0.71 | 5.60±1.14 |
|  | 30 | 5.08±0.93 | 5.03±1.01 | 5.00±1.04 | 5.67±1.34 |
| **EO (x10^3^/ul)** | 0 | 0.45±0.29 | 0.43±0.26 | 0.50±0.22 | 0.28±0.23 |
|  | 30 | 0.48±0.18 | 0.48±0.49 | 0.50±0.47 | 0.48±0.25 |
| **RBC (x10^6^/ul)** | 0 | 6.29±0.77 | 6.65±0.39 | 6.70±0.31 | 6.75±0.81 |
|  | 30 | 6.66±0.49 | 6.71±0.57 | 6.66±0.51 | 6.69±0.53 |
| **HGB (g/l)** | 0 | 154.62±11.02 | 157.50±9.47 | 155.00±16.43 | 157.50±18.06 |
|  | 30 | 155.83±12.06 | 159.25±12.01 | 158.25±10.56 | 154±13.36 |
| **HCT (%)** | 0 | 46.42±2.48 | 45.93±3.69 | 47.70±5.72 | 50.63±1.27 |
|  | 30 | 45.43±3.85 | 46.75±3.95 | 46.28±4.79 | 47.23±3.73 |
| **PLT (x10^3^/ul)** | 0 | 503.55±185.19 | 508.00±75.18 | 511.25±73.48 | 450.17±119.63 |
|  | 30 | 489.50±136.32 | 547.75±56.33 | 522.50±31.86 | 401.50±132.40 |

WBC, white blood cells; LYM, lymphocytes; MO, monocytes; SN, neutrophil granulocytes; EO, eosinophilic granulocytes RBC, red blood cells; HGB, hemoglobin; HCT, hematocrit; PLT, platelets.
