# Supplementary material for: Subchronic Toxicity of the New Iodine Complex in Dogs and Rats
Source: Front Vet Sci. 2020 Apr 17;7:184. doi: 10.3389/fvets.2020.00184 (PMC7181231; doi:10.3389/fvets.2020.00184)
Supplement: Supplementary file 7 [file Table_7.DOCX]

Table S7. Data from serum clinical chemistry tests in male dogs

| **Parameter** | **Dose, mg/kg** | | | | | | | |
| --- | --- | --- | --- | --- | --- | --- | --- | --- |
|  | **Vehicle (water)** | | **30** | | **75** | | **180** | |
|  | **Day of study** | | | | | | | |
|  | **0** | **30** | **0** | **30** | **0** | **30** | **0** | **30** |
| **ALT ukat/l** | 0.58 ± 0.10 | 0.55 ± 0.09 | 0.77 ± 0.47 | 0.77 ± 0.47 | 0.48 ± 0.05 | 0.50 ± 0.05 | 0.57 ± 0.17 | 0.46 ± 0.12 |
| **AST ukat/l** | 0.47 ± 0.08 | 0.46 ± 0.10 | 0.46 ± 0.11 | 0.46 ± 0.11 | 0.40 ± 0.05 | 0.39 ± 0.06 | 0.52 ± 0.11 | 0.48 ± 0.05 |
| **ALP ukat/l** | 1.34 ± 0.22 | 1.22 ± 0.32 | 1.17 ± 0.14 | 1.17 ± 0.14 | 1.37 ± 0.15 | 1.42 ± 0.12 | 1.13 ± 0.20 | 1.87 ± 0.51 |
| **Chol mmol/l** | 5.23 ± 1.10 | 5.01 ± 0.92 | 5.07 ± 0.54 | 5.07 ± 0.54 | 5.11 ± 0.64 | 4.81 ± 0.64 | 4.97 ± 1.14 | 6.01 ± 1.41 |
| **TP g/l** | 60.73 ± 1.67 | 60.45 ± 3.00 | 62.50 ± 4.12 | 62.50 ± 4.12 | 57.75 ± 1.50 | 56.50 ± 2.65 | 59.67 ± 8.96 | 56.67 ± 6.06 |
| **Alb g/l** | 12.28 ± 0.57 | 12.67 ± 1.03 | 14.00 ± 0.82 | 14.00 ± 0.82 | 12.75 ± 1.26 | 12.25 ± 1.50 | 12.83 ± 1.94 | 10.33 ± 2.25 |
| **Glo g/l** | 48.45 ± 1.36 | 47.78 ± 2.90 | 48.50 ± 3.87 | 48.50 ± 3.87 | 45.00 ± 1.41 | 44.25 ± 1.50 | 46.02 ± 7.07 | 46.00 ± 4.86 |
| **Glu mmol/l** | 4.90 ± 0.19 | 4.75 ± 0.48 | 4.98 ± 0.36 | 5.03 ± 0.43 | 4.90 ± 0.14 | 4.88 ± 0.19 | 4.48 ± 0.28 | 4.47 ± 0.45 |
| **Urea mmol/l** | 4.20 ± 0.95 | 4.07 ± 1.03 | 3.73 ± 0.22 | 3.73 ± 0.22 | 4.23 ± 0.88 | 4.23 ± 0.82 | 4.18 ± 1.11 | 3.45 ± 0.92 |
| **Crea umol/l** | 44.33 ± 14.88 | 48.67 ± 8.48 | 52.75 ± 3.69 | 52.75 ± 3.69 | 42.25 ± 4.35 | 39.50 ± 4.62 | 50.83 ± 5.78 | 45.00 ± 8.67 |
| **Bbn umol/l** | 6.00 ± 0.00 | 6.00 ± 0.00 | 6.00 ± 0.00 | 6.00 ± 0.00 | 6.00 ± 0.00 | 6.00 ± 0.00 | 6.00 ± 0.00 | 6.00 ± 0.00 |
| **Na mmol/l** | 148.10 ± 2.31 | 147.17 ± 1.18 | 146.50 ± 2.08 | 149.50 ± 2.08 | 149.50 ± 2.08 | 150.00 ± 1.63 | 147.17 ± 2.99 | 148.83 ± 2.32 |
| **K mmol/l** | 4.70 ± 0.26 | 4.75 ± 0.21 | 4.30 ± 0.42 | 4.30 ± 0.42 | 4.38 ± 0.32 | 4.50 ± 0.14 | 4.64 ± 0.40 | 4.72 ± 0.40 |
| **Cl mmol/l** | 114.12 ± 2.79 | 113.33 ± 1.37 | 112.75 ± 2.75 | 112.75 ± 2.75 | 113.75 ± 3.59 | 114.50 ± 3.87 | 113.17 ± 2.99 | 114.67 ± 2.73 |

ALT, alanine aminotransferase; AST, aspartate aminotransferase; ALP, alkaline phosphatase; Bbn, bilirubin; Alb, albumin; Glo, globulins; TP, total protein; Glu, glucose; Urea, urea; Crea, creatinine; Chol, cholesterol; Na, sodium; K, potassium; Cl, chlorides.
