# Supplementary material for: Subchronic Toxicity of the New Iodine Complex in Dogs and Rats
Source: Front Vet Sci. 2020 Apr 17;7:184. doi: 10.3389/fvets.2020.00184 (PMC7181231; doi:10.3389/fvets.2020.00184)
Supplement: Supplementary file 8 [file Table_8.DOCX]

Table S8. Data from serum clinical chemistry tests in female dogs

| **Parameter** | **Dose, mg/kg** | | | | | | | |
| --- | --- | --- | --- | --- | --- | --- | --- | --- |
|  | **Vehicle (water)** | | **30** | | **75** | | **180** | |
|  | **Day of study** | | | | | | | |
|  | **0** | **30** | **0** | **30** | **0** | **30** | **0** | **30** |
| **ALT ukat/l** | 0.56 ± 0.09 | 0.56 ± 0.08 | 0.50 ± 0.08 | 0.53 ± 0.06 | 0.52 ± 0.05 | 0.56 ± 0.10 | 0.61 ± 0.17 | 0.44 ± 0.11 |
| **AST ukat/l** | 0.48 ± 0.03 | 0.44 ± 0.07 | 0.46 ± 0.07 | 0.42 ± 0.04 | 0.42 ± 0.07 | 0.43 ± 0.06 | 0.48 ± 0.10 | 0.51 ± 0.11 |
| **ALP ukat/l** | 1.31 ± 0.35 | 1.38 ± 0.35 | 1.18 ± 0.07 | 1.28 ± 0.12 | 1.32 ± 0.24 | 1.39 ± 0.10 | 1.33 ± 0.28 | 1.78 ± 0.66 |
| **Chol mmol/l** | 5.56 ± 1.03 | 5.48 ± 0.96 | 4.89 ± 0.25 | 5.44 ± 0.27 | 4.82 ± 0.72 | 5.10 ± 0.47 | 5.25 ± 0.24 | 5.94 ± 1.81 |
| **TP g/l** | 59.62 ± 4.32 | 60.33 ± 1.86 | 58.50 ± 1.91 | 58.25 ± 2.36 | 56.75 ± 2.99 | 59.00 ± 2.31 | 60.33 ± 2.07 | 57.00 ± 6.93 |
| **Alb g/l** | 12.38 ± 0.85 | 13.72 ± 0.78 | 13.00 ± 1.41 | 12.50 ± 1.29 | 12.25 ± 0.96 | 13.50 ± 1.00 | 12.83 ± 0.75 | 10.33 ± 2.25 |
| **Glo g/l** | 46.43 ± 3.82 | 46.62 ± 2.00 | 45.50 ± 0.58 | 45.75 ± 1.89 | 44.50 ± 2.89 | 46.25 ± 1.71 | 46.33 ± 1.21 | 46.67 ± 6.65 |
| **Glu mmol/l** | 4.92 ± 0.32 | 4.77 ± 0.22 | 5.28 ± 0.31 | 4.85 ± 0.24 | 5.05 ± 0.13 | 5.00 ± 0.14 | 4.52 ± 0.48 | 4.37 ± 0.29 |
| **Urea mmol/l** | 3.38 ± 0.37 | 3.90 ± 0.59 | 3.83 ± 0.39 | 3.90 ± 0.87 | 4.63 ± 0.85 | 4.43 ± 0.37 | 3.67 ± 1.08 | 3.50 ± 0.46 |
| **Crea umol/l** | 49.35 ± 8.66 | 50.33 ± 6.11 | 49.68 ± 13.97 | 48.18 ± 7.21 | 42.00 ± 2.71 | 44.75 ± 3.10 | 45.50 ± 1.37 | 50.17 ± 8.06 |
| **Bbn umol/l** | 6.00 ± 0.00 | 6.00 ± 0.00 | 6.00 ± 0.00 | 6.00 ± 0.00 | 6.00 ± 0.00 | 6.00 ± 0.00 | 6.00 ± 0.00 | 6.00 ± 0.00 |
| **Na mmol/l** | 148.38 ± 2.00 | 149.27 ± 0.98 | 146.50 ± 1.91 | 149.33 ± 0.94 | 148.50 ± 4.51 | 150.50 ± 2.38 | 146.50 ± 2.35 | 147.67 ± 1.37 |
| **K mmol/l** | 4.67 ± 0.24 | 4.65 ± 0.47 | 4.53 ± 0.33 | 4.55 ± 0.24 | 4.08 ± 0.39 | 4.13 ± 0.22 | 4.37 ± 0.37 | 4.73 ± 0.33 |
| **Cl mmol/l** | 113.67 ± 2.34 | 115.72 ± 0.94 | 113.75 ± 2.50 | 114.75 ± 1.50 | 112.25 ± 4.11 | 114.25 ± 2.22 | 11.67 ± 2.58 | 115.00 ± 1.67 |

ALT, alanine aminotransferase; AST, aspartate aminotransferase; ALP, alkaline phosphatase; Bbn, bilirubin; Alb, albumin; Glo, globulins; TP, total protein; Glu, glucose; Urea, urea; Crea, creatinine; Chol, cholesterol; Na, sodium; K, potassium; Cl, chlorides.
