# Supplementary figures and images for: Subchronic Toxicity of the New Iodine Complex in Dogs and Rats
Source: Front Vet Sci. 2020 Apr 17;7:184. doi: 10.3389/fvets.2020.00184 (PMC7181231; doi:10.3389/fvets.2020.00184)

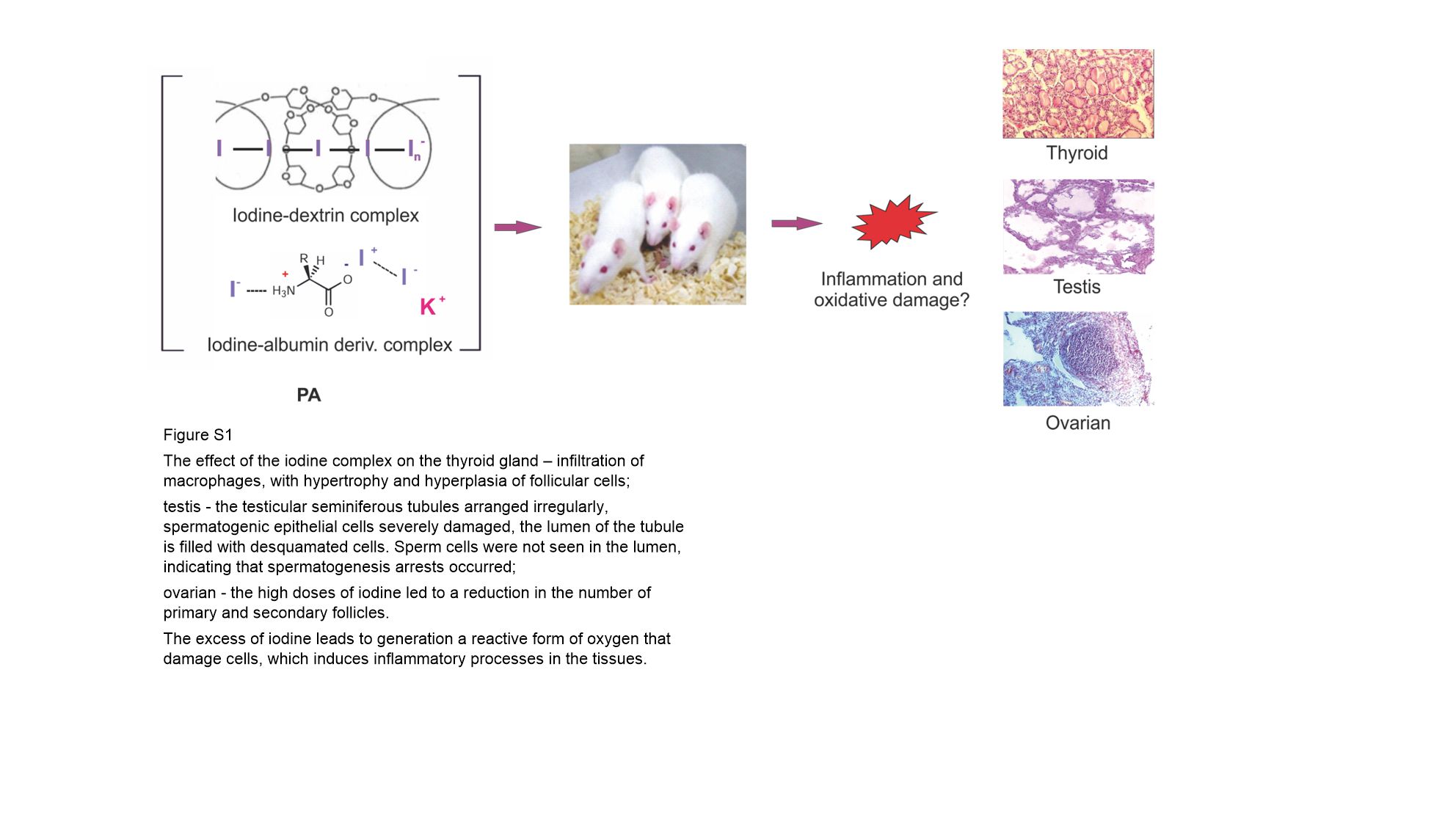

Supplement: Supplementary file 9 [file Image_1.jpg]
